# Vegetative Characters, Growth Habit and Microsporangiate Strobilus of Lycopsid *Minostrobus chaohuensis*

**DOI:** 10.1371/journal.pone.0122167

**Published:** 2015-03-27

**Authors:** Mei-Cen Meng, De-Ming Wang, Jian-Xin Yao

**Affiliations:** 1 Ministry of Land and Resources Key Laboratory of Stratigraphy and Paleontology, Institute of Geology, Chinese Academy of Geological Sciences, Beijing, China; 2 Key Laboratory of Orogenic Belts and Crustal Evolution, Department of Geology, Peking University, Beijing, China; Institute of Botany, CHINA

## Abstract

Late Devonian *Minostrobus chaohuensis* is one of the earliest monosporangiate-strobilate isoetaleans. Based on new material of this plant, the vegetative axis and microsporangiate strobilus are studied in detail, and the whole plant knowledge is summarized. The vegetative axis is isotomously branched. The stem is up to 55 mm in diameter with helically arranged leaf cushions. Stems and thick branches bear long fusiform leaf cushions and interareas with vertical linear ornamentations. A ligule pit, oblanceolate leaf scar, and vascular bundle scar appear on the leaf cushion. Distal axes have persistent lanceolate leaves and rhombic leaf bases. The microsporangiate strobilus is cylindrical in shape, possesses sporophyll with alate pedicel and long triangular lamina, uniseriate sporangial wall, subarchesporial pad inside the sporangium, and microspore with cingulum. Based on comparisons with other isoetaleans, the usage of the terms “leaf cushion” and “leaf base” is discussed, and *Minostrobus chaohuensis* is considered as a tree-like lycopsid. It suggests that arborescent isoetaleans with monosporangiate strobili had appeared and diversified in the Late Devonian. The multi-dichotomous branching system of *Minostrobus* provides new data on the evolution of growth architecture in rhizomorphic lycopsids.

## Introduction

Arborescent lycopsids of Isoëtales *sensu lato* Meyen, especially the relatively derived clade with monosporangiate strobili (Dichostrobiles DiMichele and Bateman), are the most conspicuous plants of the Carboniferous landscape around the world [1], [2]. *Sublepidodendron* (Nathorst) Hirmer as a member of Dichostrobiles flourished in the Late Devonian of South China [3–5]. In this study, another isoetalean with monosporangiate strobili, *Minostrobus* Wang, is suggested to have arborescent habit. Detailed research on this plant adds to our knowledge of the evolution of lycopsids in the Devonian.

*Minostrobus chaohuensis* Wang was reported from the Upper Devonian Wutong (Wutung) Formation of Chaohu City, Anhui Province, South China, and was established on the basis of strobili containing megaspores [6]. Subsequent studies revealed the megasporangiate strobilus characters in detail and assigned this plant to Dichostrobiles [7, 8]. Although the vegetative axis and microsporangiate strobilus have been described [8], some important characters are still not clear. Now, we obtained some well-preserved specimens containing permineralized microsporangiate strobili from the same formation and locality. Based on the new material and sections, we emend the morphology of vegetative leaf, leaf cushion and microspore, describe the anatomy of microsporangiate strobilus. We also debate the usage of terms “leaf cushion” and “leaf base” among lycopsids, discuss the growth habit of *M*. *chaohuensis* and its evolutionary significance.

## Materials and Methods

New material was collected from the Fenghuangshan Section, about 3.0 km north of Chaohu City, Anhui Province, China. The specific location of this section (31°37′51″N and 117°50′54″E) was illustrated by Meng et al. [7]. The fossil-bearing bed belongs to the middle part of the Leigutai Member (upper member of the Wutong Formation). This stratum includes lower and upper clay layers intercalated with quartz sandstone, which represents the boundary between Devonian and Carboniferous sediments based on spore, plant and invertebrate assemblage [9]. Specimens in this study are the latest Devonian in age, some of them occur in the same layer with those described by Meng et al. [7], while the others were collected from a higher horizon. No specific permission was required for the field study in the location, and the field study did not involve endangered or protected species.

The plant is mostly preserved as impressions and compressions in black-gray mudstone, with some strobili permineralized by limonite. We used steel needles to expose the morphology. The permineralized microsporangiate strobili were embedded in epoxy resin, sectioned, grinded, and polished to make slides for examination under light microscopy (LM). Before embedding, we selected masses of microspores for observation under scanning electron microscopy (SEM). All specimens and slides are housed at Department of Geology, Peking University, Beijing, China.

## Results

### Description

**Fig 1:**
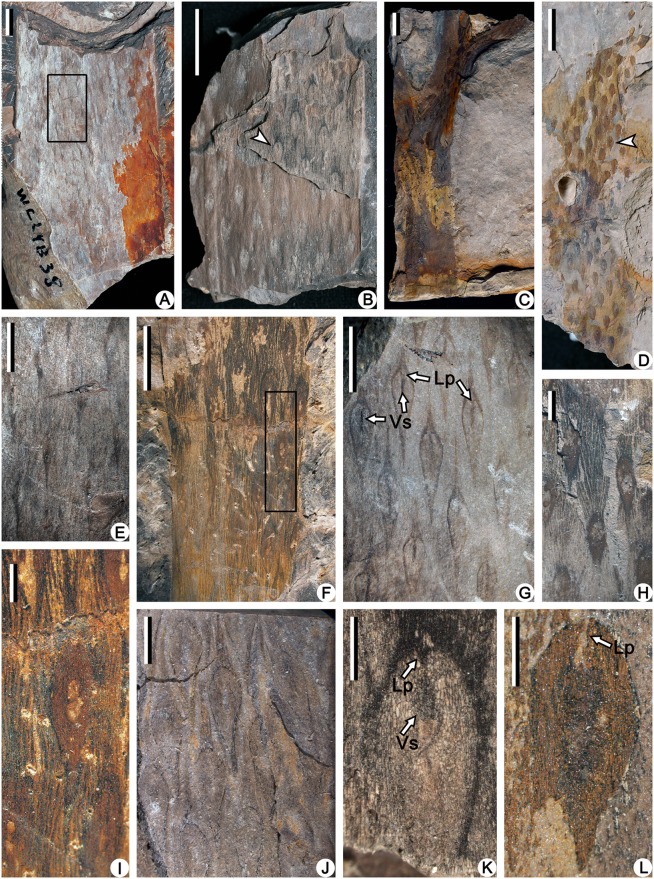
Compressions of vegetative axes of *Minostrobus chaohuensis*. (A) The widest stem. Rectangle indicating portion enlarged in Fig. 1E. PKUB12138. Scale bar = 10 mm. (B) Stem with the lower left part without epidermis, showing leaf cushions. Arrow indicating portion enlarged in Fig. 1K. PKUB12145. Scale bar = 10 mm. (C) Stem dichotomizing two times. PKUB12101. Scale bar = 10 mm. (D) Vegetative axis with leaf cushions arranged in helix. Arrow indicating portion enlarged in Fig. 1L. PKUB12137. Scale bar = 10 mm. (E) Enlargement of Fig. 1A (rectangle), showing parastichies of leaf cushions. PKUB12138. Scale bar = 5 mm. (F) Vegetative axis with leaf cushions arranged in helix. Rectangle indicating portion enlarged in Fig. 1I. PKUB12135. Scale bar = 5 mm. (G) Vegetative axis with long fusiform leaf cushions, showing leaf scars, ligule pits (Lp) and vascular scars (Vs). PKUB12171. Scale bar = 5 mm. (H) Vegetative axis showing interareas with vertical linear ornamentations among leaf cushions. PKUB12163. Scale bar = 2 mm. (I) Enlargement of Fig. 1F (rectangle), showing long fusiform leaf cushion and ornamentations on the interareas. PKUB12135. Scale bar = 1 mm. (J) Vegetative axis with leaf cushions, showing the oblanceolate leaf scars. PKUB12148. Scale bar = 2 mm. (K) Enlargement of Fig. 1B (arrow), showing leaf scar, ligule pit (Lp) and vascular scar (Vs). PKUB12145. Scale bar = 1 mm. (L) Enlargement of Fig. 1D (arrow), showing leaf scar and ligule pit (Lp). PKUB12137. Scale bar = 1 mm.

**Fig 2:**
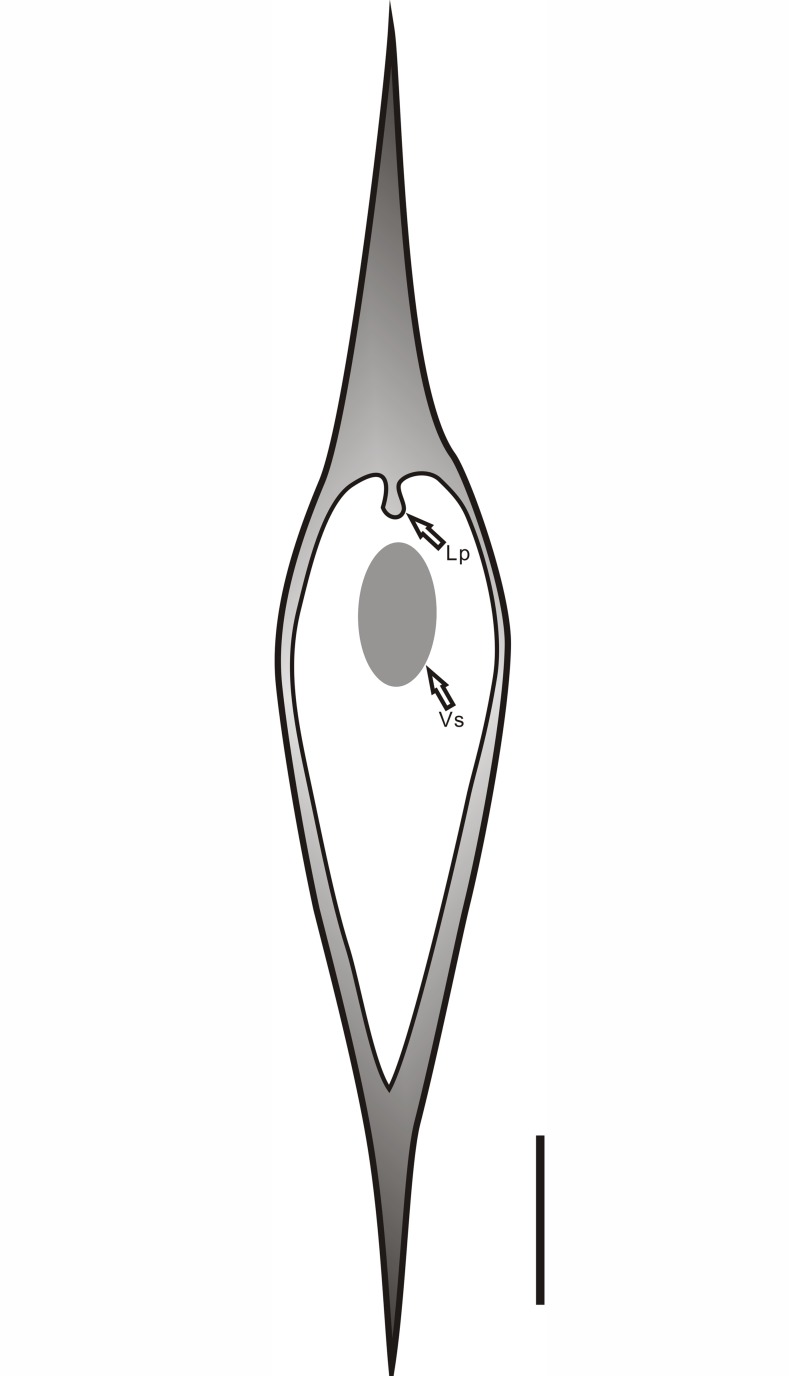
Diagram of the leaf cushion of *Minostrobus chaohuensis*. Oblanceolate leaf scar, ligule pit (Lp) and vascular scar (Vs). Scale bar = 1 mm.

**Fig 3:**
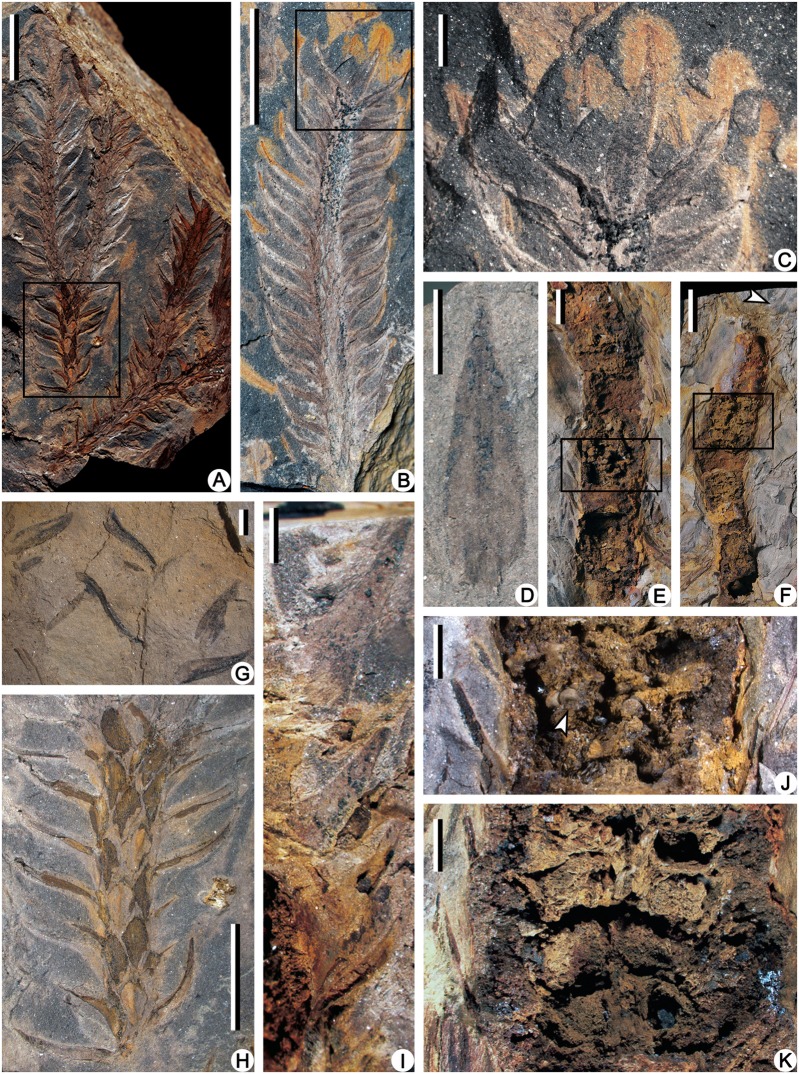
Compressions of leafy axes and leaves (A-D, G, H), limonite permineralized strobili (E, F, I-K) of *Minostrobus chaohuensis*. (A) Dichotomous vegetative axes with persistent leaves. Rectangle indicating portion enlarged in Fig. 3H. PKUB12124. Scale bar = 10 mm. (B) Distal part of vegetative axis with persistent leaves. Rectangle indicating portion enlarged in Fig. 3C. PKUB12139. Scale bar = 10 mm. (C) Enlargement of Fig. 3B (rectangle), showing the lanceolate leaf in face view. PKUB12139. Scale bar = 2 mm. (D) Lanceolate vegetative leaf in face view. PKUB12159. Scale bar = 2 mm. (E) Limonite permineralized megasporangiate strobilus. Rectangle indicating portion enlarged in Fig. 3J. PKUB12188. Scale bar = 5 mm. (F) Limonite permineralized microsporangiate strobilus. Arrow indicating portion enlarged in Fig. 3I. Rectangle indicating portion enlarged in Fig. 3K. PKUB12189. Scale bar = 10 mm. (G) Detached vegetative leaves in the matrix. PKUB12160. Scale bar = 2 mm. (H) Enlargement of Fig. 3A (rectangle), showing vegetative leaves in lateral view and rhombus leaf bases. PKUB12124. Scale bar = 5 mm. (I) Enlargement of Fig. 3F (arrow), showing laminae of microsporophyll. PKUB12189. Scale bar = 2 mm. (J) Enlargement of Fig. 3E (rectangle). Arrow indicating megaspores. PKUB12188. Scale bar = 2 mm. (K) Enlargement of Fig. 3F (rectangle), showing strobilar axis and microsporangia on the adaxial surface of sporophyll pedicel. PKUB12189. Scale bar = 2 mm.

The stems are 25–55 mm wide (Fig. 1A–C), and branch isotomously at the top (Fig. 1C). Long fusiform leaf cushions are 6.0–9.0 mm long and 1.0–1.6 mm wide (Fig. 1B, F, G, I, J), helically arranged on stems and branches that are at least 8.0 mm wide. Parastichies cross at nearly right angles, whereas orthostichy and horizontal rows are absent (Fig. 1A–G). Evident interareas with vertical linear ornamentations among the leaf cushions exist on axes thicker than 10 mm (Fig. 1B, F, H), and their area is in proportion to the width of axes (Fig. 1A, C, F). An oblanceolate leaf scar is located at the middle part of the leaf cushion, occupying half the length and almost all the width of the cushion (Figs. 1B, G, I, J and 2). An evident and depressed ligule pit (Lp) appears on the top of the leaf scar, and an oval or linear vascular bundle scar (Vs) exists on the upper part of the leaf scar (Figs. 1B, G, K and 2). In some specimens, the leaf cushion can’t be recognized but the oblanceolate leaf scar and depressed ligule pit (Lp) were observed (Fig. 1B, D, L). Distal axes are 2.0–3.0 mm wide, with persistent vegetative leaves arranged helically (Fig. 3A, B, H). The leaf is inserted at acute angle onto the axis, with a decurrent base and a linear profile in lateral view (Fig. 3A, B, H). From face view, however, the leaf is lanceolate in outline, 5.0–7.0 mm long and about 1.5 mm wide at the base (Fig. 3B–D, G). Leaf bases were exposed when the leaves broke off, and they are rhombic in shape, adjacent with each other, and about 2.5 mm long and 1.3 mm wide (Fig. 3H).

**Fig 4:**
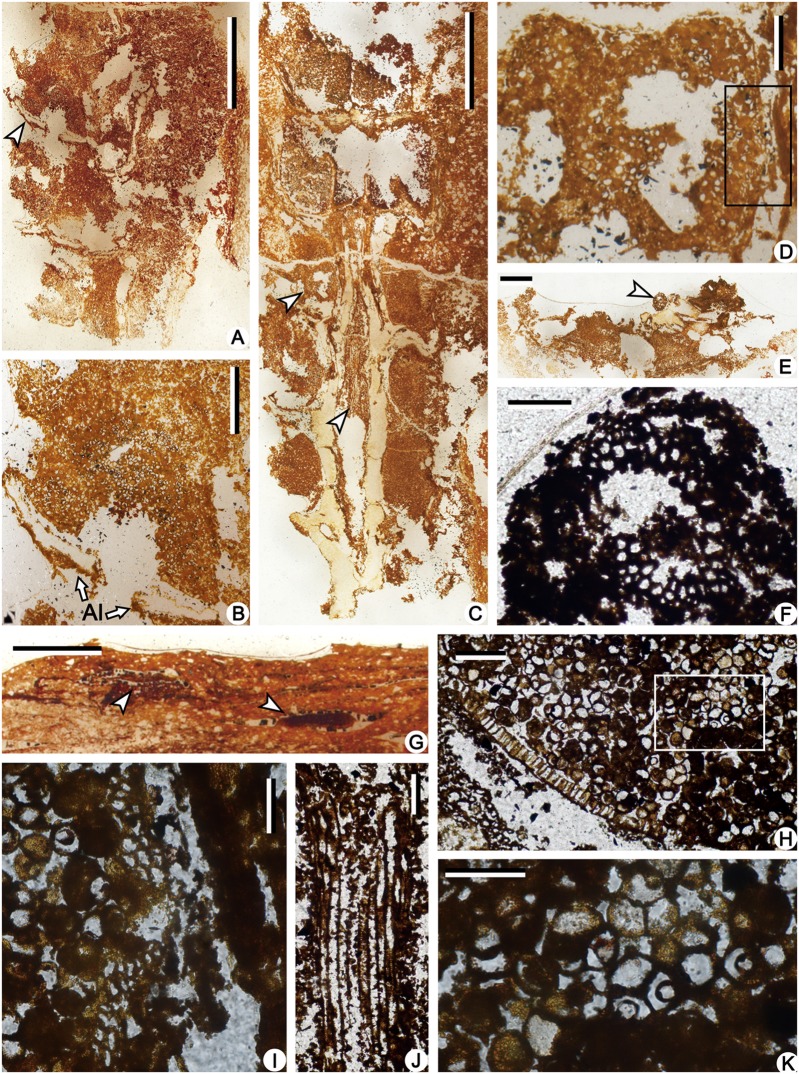
Sections of the permineralized microsporangiate strobilus of *Minostrobus chaohuensis* in Fig. 3F, under LM. (A) Longitudinal section of strobilus, showing sporophylls and sporangia. Arrow indicating portion enlarged in Fig. 4B. PKUB12189-3-4. Scale bar = 2 mm. (B) Enlargement of Fig. 4A (arrow), showing microsporangium with numerous microspores and alations (Al) of sporophyll. PKUB12189-3-4. Scale bar = 0.5 mm. (C) Radial section of strobilus, showing strobilar axis, sporophylls and microsporangia. Arrows indicating portions enlarged in Fig. 4D and Fig. 4J. PKUB12189-3-3. Scale bar = 2 mm. (D) Enlargement of Fig. 4C (upper arrow), showing sporangium containing microspores and subarchesporial pad. Rectangle indicating portion enlarged in Fig. 4I. PKUB12189-3-3. Scale bar = 0.2 mm. (E) Transverse section of strobilus, showing strobilar axis and microsporangia. Arrow indicating portion enlarged in Fig. 4F. PKUB12189-2-3. Scale bar = 1 mm. (F) Enlargement of Fig. 4E (arrow), showing exarch primary xylem of strobilar axis. PKUB12189-2-3. Scale bar = 0.1 mm. (G) Transverse section of strobilus. Arrows indicating transverse view of sporophylls. PKUB12189-1-4. Scale bar = 1 mm. (H) Transverse section of uniseriate sporangial wall, showing columnar cells and microspores in the sporangium. Rectangle indicating portion enlarged in Fig. 4K. PKUB12189-2-5. Scale bar = 0.1 mm. (I) Enlargement of Fig. 4D (rectangle), showing subarchesporial pad and microspores in sporangium. PKUB12189-3-3. Scale bar = 50 μm. (J) Enlargement of Fig. 4C (lower arrow), showing xylem strand of strobilar axis and metaxylem tracheids with scalariform thickenings. PKUB12189-3-3. Scale bar = 0.1 mm. (K) Enlargement of Fig. 4H (rectangle), showing microspore tetrads and the inner body. PKUB12189-2-5. Scale bar = 50 μm.

**Fig 5:**
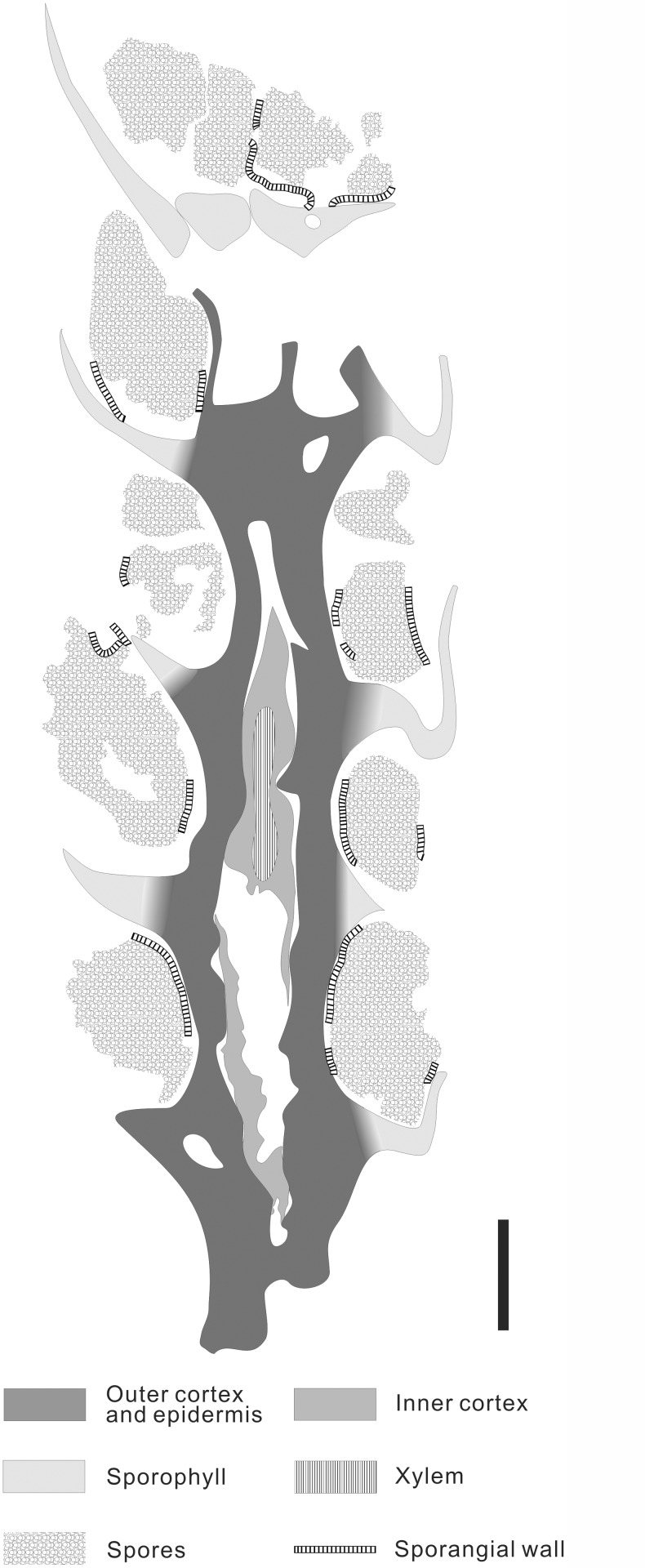
Line drawing of Fig. 4C. Radial section of microsporangiate strobilus of *Minostrobus chaohuensis*. Scale bar = 1 mm.

**Fig 6:**
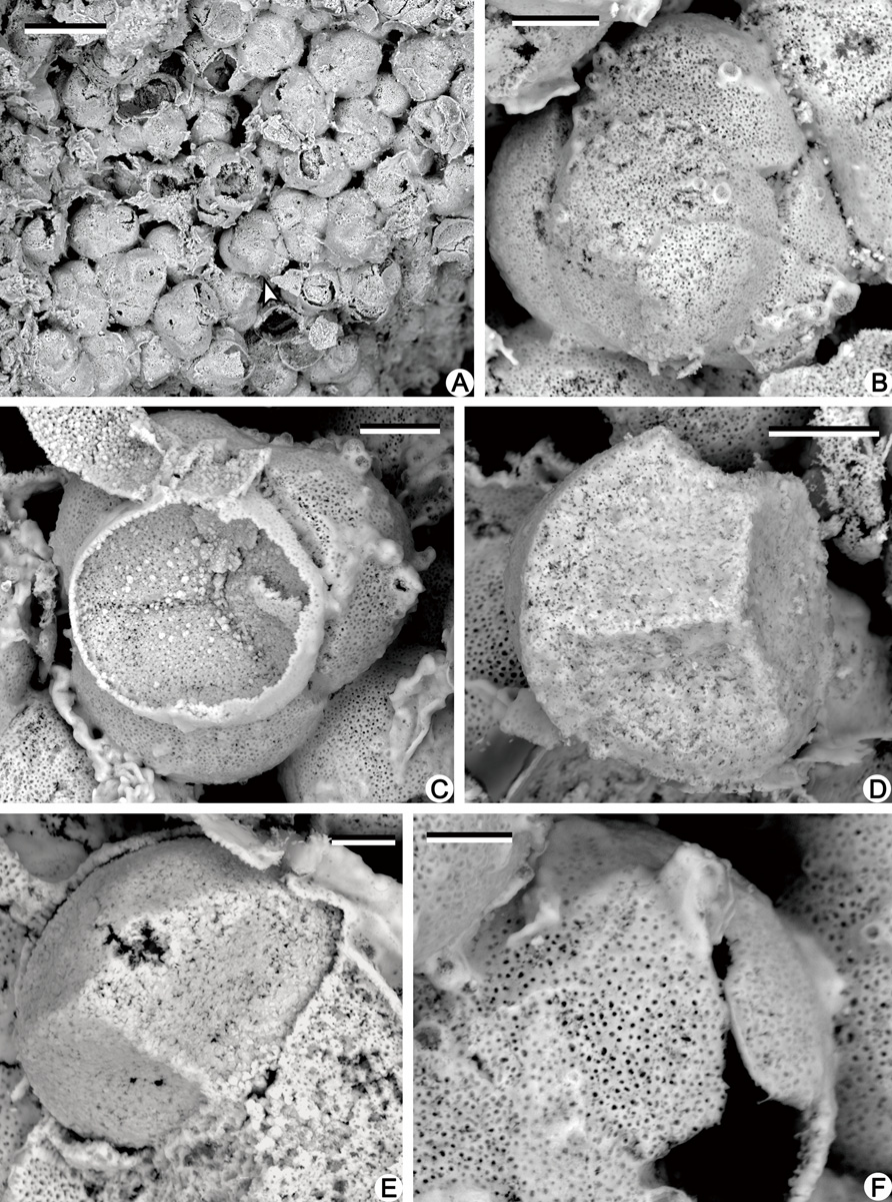
SEM observations of microspores of *Minostrobus chaohuensis*. (A) Tetrads of microspores. Arrow indicating portion enlarged in Fig. 6B. Scale bar = 50 μm. (B) Enlargement of Fig. 6A (arrow), showing a tetrad with four visible microspores. Scale bar = 10 μm. (C) A tetrad with one of the microspores broken in the distal face. Scale bar = 10 μm. (D) A microspore showing the trilete and cingulum. Scale bar = 10 μm. (E) A microspore showing the inner and outer exospores. Scale bar = 5 μm. (F) Foveolate outer exospore of the microspore. Scale bar = 5 μm.

The permineralized strobili are cylindrical in shape and slightly curved, with both ends missing (Fig. 3E, F). The megasporangiate strobilus is about 40 mm long and has megaspores preserved (Fig. 3E, J, arrow). The microsporangiate strobilus is about 30 mm long and 6.0 mm wide excluding distal part of sporophylls (Fig. 3F). The strobilar axis is about 1.0 mm in diameter, with an exarch primary xylem about 0.3 mm in diameter (Figs. 3K; 4C, E, F and 5). The protoxylem tracheids are about 5.0 μm in diameter, and the metaxylem tracheids with scalariform thickenings are about 20 μm in diameter (Fig. 4F, J). The microsporophyll pedicel is about 2.5 mm in length, perpendicular to the strobilar axis (Fig. 3K), and expands laterally to be at least 1.7 mm wide and form alations (Al, Fig. 4A, B). The upturned lamina is long triangular in outline, about 5.0 mm long and 3.0 mm wide at the base (Fig. 3I). In transverse section, the lamina is about 0.1–0.2 mm thick and tapers laterally (Fig. 4G, arrows). Single spherical to ellipsoidal microsporangium, about 2.5 mm long and 1.5 mm high, occurs on the adaxial surface of the sporophyll pedicel (Figs. 3K; 4A–C; 5). The sporangial wall is about 25 μm thick and consists of a single layer of columnar cells (Figs. 4H and 5). Inside the wall, subarchesporial pad containing polygonal parenchyma cells can be observed (Fig. 4D, I). The parenchyma cells are 10–15 μm in diameter and associated with microspores (Fig. 4D, I). Each microsporangium contains numerous microspores (Figs. 4A–D and 5). Four microspores in a tetrad configuration are arranged tetrahedrally (Figs. 4H, K and 6A–C). The trilete microspores with narrow cingulum are 20–30 μm in diameter and circular in equatorial shape (Figs. 4K and 6B–D). Round inner body exists in some microspores (Fig. 4I, K). The exospore with two layers (Fig. 6E) is 2.0–4.0 μm thick (Fig. 4K). No ornamentation was observed on the foveolate surface of outer exospore (Fig. 6F), possibly resulted by leaching. The microspores are similar with *Lycospora* (Ibrahim) Schopf et al. in size and existence of cingulum [10].

### Systematics

Class: Lycopsida Pichi-Sermolli 1958

Order: Isoёtales *sensu lato* Meyen 1987

Suborder: Dichostrobiles DiMichele et Bateman 1996

Family: Incertae sedis

Genus: *Minostrobus* Wang 2001 emend.

Emended diagnosis: Arborescent lycopsid with monosporangiate-strobilus, possibly monoecious. Aerial axes multi-dichotomous. Leaf with single mid-vein abscised from stem and thick branch. Long fusiform leaf cushion with ligule pit and oblanceolate leaf scar. Among leaf cushion with evident interareas possessing linear ornamentations. Axis with solid exarch primary xylem. Protoxylem confined to ridges at periphery of primary xylem strands. Metaxylem tracheids bearing Williamson’s striations. Single spherical to elliptical sporangium inserted to adaxial side of sporophyll with smooth margin, pointed apex and alate pedicel. Sporangial wall comprising one layer of columnar cells. Subarchesporial pad along inner surface of sporangial wall. Megasporangium connected to sporophyll by narrow attachment, with sporangial base surrounded by alations. Each megasporangium with four megaspores.

Type species: *Minostrobus chaohuensis* Wang 2001 emend.

Holotype: PB18751 (Fig. 8 in Wang, 2001 [6])

Type locality: The south slope of Beishan hill, Shizikou Section, 3 km north of Chaohu City, Anhui Province, China.

Repository: Nanjing Institute of Geology and Palaeontology, Chinese Academy of Sciences, Nanjing, China.

Stratigraphy: Leigutai Member of the Wutong Formation.

Specimens examined herein: PKUB12101, PKUB12124, PKUB12135, PKUB12137–PKUB12139, PKUB12145, PKUB12148, PKUB12159, PKUB12160, PKUB12163, PKUB12171, PKUB12188 and PKUB12189 (see Figs. 1, 3, 4, and 6).

Repository: Department of Geology, Peking University, Beijing, China.

Locality: Fenghuangshan Section, Chaohu City, Anhui Province, China.

Stratigraphy: Leigutai Member of the Wutong Formation.

Age: Late Devonian (Famennian).

Emended diagnosis: As for generic diagnosis. Aerial axes dichotomizing at least eight times. Lanceolate sterile leaves with smooth margin. Long fusiform leaf cushions about 6.0–9.0 mm long and 1.0–1.6 mm wide, with oblanceolate leaf scar located at the middle part and occupying half the length and almost all the width of the cushion. Ligule pit located on the top of leaf scar. Axis with single, solid exarch primary xylem. Twelve protoxylem ridges with annular tracheids around the metaxylem core. Polygonal metaxylem tracheids about 20–40 μm in diameter, with scalariform and reticulate thickenings. Cortical cells rectangular in outline. Separate monosporangiate strobili attached at apices of the axes. Microsporangiate strobilus over 80 mm long and about 6.0 mm wide. Microsporophyll pedicel about 2.5 mm long, with alations up to 1.7 mm wide. Long triangular upturned lamina about 5.0 mm long and 3.0 mm wide at the base. Smooth microsporangium about 2.5 mm long and 1.5 mm high. Microspores *Lycospora*-type, about 20–30 μm in diameter, with circular amb, inner body and narrow cingulum. Microspore exospore with two layers. Megasporangiate strobilus about 5.0 mm wide. Megasporophyll trace arising from stele at acute angles. Megasporophylls arranged in 2/9 helix, with angles between parastichies and horizontal line being about 35°. Pedicel about 1.8 mm long, proximally “V” form in transverse view, distally expanding to form alations. Lamina long-triangular in face view. Smooth megasporangium about 2.0 mm long, 1.5 mm wide and 1.5 mm high. Attachment between megasporangium and megasporophyll about 1.0 mm long and 0.2 mm wide. Megaspores *Lagenicula*-type, from about 370 μm to about 1490 μm in diameter, with distinct gula and spiny ornamentation, more or less circular amb, pear shape in equatorial view. Spiny ornamentation about 20 μm wide at base. Megaspore wall with thin inner exospore and spongy outer exospore.

### Comparisons with *Minostrobus chaohuensis* described by Wang et al. (2012)

Material in this study was collected over areas of several square meters from two bedding planes. In these areas, only one type of stem, branch, leafy axis, and microsporangiate strobilus were found. Therefore, they most probably represent portions of a single type of lycopsid. In addition, these fossils are preserved together with megasporangiate strobili of *Minostrobus chaohuensis*, and similar with *M*. *chaohuensis* described by Wang et al. [8] in characters as follows: 1) size and branching pattern of axis; 2) length of vegetative leaf and width of leaf cushion; 3) size and type of microspore (Table 1). We thus conclude that they are assignable to *M*. *chaohuensis*. However, some important characters of vegetative axis and microsporangiate strobilus are emended (Table 1). Wang et al. considered *M*. *chaohuensis* as a lycopsid with persistent linear vegetative leaves, and interpreted the fusiform structure on the axis as “leaf base” [8]. From evident leaf scars on the axis and scattered leaves in the matrix, we deduce that vegetative leaves on wider axes have abscised and those fusiform structures should be defined as “leaf cushion”. Based on face view of the vegetative leaf and microsporophyll lamina, we emend their shape as lanceolate and long triangular, respectively. Besides, we recognize some new vegetative and fertile traits of this plant, e.g., existence of ligule pit, leaf scar, microsporophyll alations, and subarchesporial pad in the microsporangium. Microsporangium and microspore with their wall structures/layers have also been discovered and clearly illustrated (Table 1).

**Table 1:** Comparisons of main characters of *Minostrobus chaohuensis* (vegetative axis and microsporangiate strobilus) described by Wang et al. [8] and this study.

	Wang et al.	This study
**Axis**	0.8–42 mm wide, dichotomize at least eight times	Stems 25–55 mm wide with isotomously branches, distal axes 2.0–3.0 mm wide
**Vegetative leaf**	Persistent	Abscised on thick axes
Shape	Linear	Lanceolate
Length (mm)	4.0–7.0	5.0–7.0
Width (mm)	0.4–0.6	About 1.5 at the base
**Leaf cushion**		
Phyllotaxy	Helically arranged	Helically arranged, parastichies crossing at right angle, orthostichies and horizontal rows absent
Shape	Wide fusiform	Long fusiform
Size (L × W, mm)	2.5–4.6 × 0.9–1.7	6.0–9.0 × 1.0–1.6
Interarea	Presence	Presence, with vertical linear ornamentations
Leaf scar	—	Oblanceolate
Ligule pit	—	Presence
Vascular scar	Presence	Presence
**Microsporangiate strobilus**		
Size (L × W, mm)	At least 80 × —	At least 30 × 6.0
Phyllotaxy	Pseudo-whorled	—
**Microsporangiate strobilar axis**		
Width (mm)	1.0–1.2	1.0
Stele	—	Exarch primary xylem about 0.3 mm in diameter
**Microsporophyll**		
Pedicel	Perpendicular to the axis	Perpendicular to the axis, about 2.5 mm long
Alations	—	Presence
Lamina	0.3–0.6 mm wide, tapers toward the apex, smooth at margin	Long triangular in shape, about 3.0 mm wide at the base, smooth at margin
**Microsporangium**		
Shape	Spherical to spherical-elliptical, no pedicel	Spherical to elliptical, without pedicel
Size	0.8–1.0 mm in diameter	About 2.5 mm long, 1.3 mm high
Sporangial wall	—	About 25 μm thick, with a single layer of columnar cells
Subarchesporial pad	—	Presence
**Microspore**		
Size	20–30 μm in diameter	20–30 μm in diameter
Type	*Lycospora*	Similar with *Lycospora*
Ornamentation	Sporadic granulated	—
Wall	—	Exospore 2.0–4.0 μm thick with two layers

Note:–, lack of information; L, length; W, width.

### Comparisons with other isoetaleans

*Longostachys latisporophyllus* Zhu et al. from the Middle Devonian (Givetian) of South China is a small arborescent lycopsid [11]. It has lobed secondary xylem and spindle-shaped leaf cushions without leaf scar. Furthermore, the linear vegetative leaves with spiny margin are much longer (20–70 mm) than those of *Minostrobus chaohuensis*.

*Leptophloeum rhombicum* Dawson has been widely reported from the Upper Devonian, and considered as a lycopsid tree with thick trunk and developed secondary tissue [12–14]. As in *Minostrobus chaohuensis*, *L*. *rhombicum* is believed to possess a ligule. Nevertheless, this plant has rhombic or fan-shaped leaf cushions, small ovate leaf scars, and thicker strobili (20–30 mm in diameter) [14].

Arborescent lycopsid *Sublepidodendron* with monosporangiate strobili was widespread from the Late Devonian to the Early Carboniferous [15–17]. Two well-studied species, *Sublepidodendron songziense* Chen and *Sublepidodendron grabaui* (Sze) Wang and Xu, occur in the Upper Devonian of South China [3, 4, 18, 19]. As in *Minostrobus chaohuensis*, both of these two species possess fusiform leaf cushions/bases and vertical ornamentations on the interareas among leaf cushion/bases. *S*. *songziense* also has *Lycospora*-type microspores and *Lagenicula*-type megaspores. However, persistent vegetative leaves of *Sublepidodendron* are linear in shape, neither ligule nor ligule pit is found, and no leaf scar presents. Different with the microsporangiate strobilus of *M*. *chaohuensis*, that of *S*. *songziense* is thicker (8.0–12 mm wide), and *S*. *grabaui* has elongate sporangia (4.0 mm long and 0.8 mm high).

*Changxingia longifolia* Wang et al. from the Upper Devonian (Famennian) of South China is an isoetalean with monosporangiate strobili possessing four *Lagenicula*-type megaspores in each megasporangium [20]. Leaf cushions of this plant and *Minostrobus chaohuensis* are similar in shape and both bear ligule pit on the top of leaf scar. However, the leaf scar of *C*. *longifolia* is oval-oblanceolate in shape, the vegetative leaf is linear, the megasporangiate strobilus is shorter (20–50 mm long), the megasporophyll is reflexed, and the megasporangium is not surrounded by sporophyll.

*Lepidostrobus* (Brongniart) Brack-Hanes and Thomas represents a group of microsporangiate strobili bearing *Lycospora*-type spores [21]. Species of this organ genus mostly occur in the Carboniferous strata, while one species—*Lepidostrobus xinjiangensis* Wang—was described from the Upper Devonian of Northwest China [22]. The microsporangiate strobilus of *Minostrobus chaohuensis* meets the definition of *Lepidostrobus* in many respects, including phyllotaxy, anatomy of axis, alations (lateral laminae) and microspore type. Nevertheless, some characteristics of *Lepidostrobus* such as heel, abaxial keel of pedicel and microsporangium attachment are not clear in *M*. *chaohuensis*.

*Lepidodendron* is one of the representative arborescent lycopsids that are widely distributed in the Carboniferous floras and persisted into the Late Permian in China [15, 23]. This genus was found to attain 40 m in height and 2.0 m in diameter [24]. *Lepidodendron* species clearly show vertically elongated leaf cushions (height-width ratio > 1) with evident leaf scar and ligule pit, and possess *Lycospora*-type microspores [25]. In contrast to *Minostrobus*, the leaf cushion is more complex (with two or four parichnos scars), and the ligule pit may be located at some distance from the leaf scar [25].

Species of *Diaphorodendron* DiMichele and *Synchysidendron* DiMichele and Bateman once included within the genus *Lepidodendron* have been separated and assigned to the Diaphorodendraceae DiMichele and Bateman [26, 27]. These plants resemble *Minostrobus* in the vertically elongate leaf cushion. In some species, e.g., *Diaphorodendron selerotecum* (Pannell) DiMichele [23, 26] and *Synchysidendron dicentricum* (Felix) DiMichele and Bateman [27–29], the aperture of ligule pit is also immediately above the leaf scar. Nevertheless, they consistently possess two parichnos scars in the leaf scar and have *Achlamydocarpon* Schumacker-Lambry type microsporangiate strobilus with *Granasporites* Alpern microspore. This kind of microsporangiate strobilus is thicker (about 13 mm in diameter) than that of *Minostrobus*, has heeled sporophyll and elongated sporangium [27, 30].

## Discussion

### Term usage of “leaf cushion” and “leaf base”

Characters of the leaf cushion (e.g., phyllotaxy, leaf scar, ligule pit, parichnos) play an important role in the classification of lycopsids, and the generally accepted definition of the term “leaf cushion” is “the lowermost part of the leaf which is usually widened and remaining on the stem after leaf abscission” [31, 32]. Correspondingly, the term “leaf scar” is defined as the abscission layer area on the leaf cushion [32]. In some cases, the leaves do not abscise but break off from the leaf bases during fracturing the rock, forming the “false leaf scar”. Such breakage has been interpreted and illustrated clearly in the description of *Archaeosigillaria* Kidston, *Tomiodendron* (Radczenko) Meyen, and *Eskdalia* Kidston (Thomas) [33–35]. Shape of the breakage changes a lot when rock is split open along different planes [33]. The false leaf scar also occurs on axes with dried and collapsed leaves, and presents as a slit on the leaf base [3, 32, 36]. The third occasion forming the false leaf scar is when the abscission layer occurs in the leaf blade instead of the attachment point [32]. On stems and thick branches of *Minostrobus chaohuensis*, we have not found any indication of persistent leaves or the basal part of leaf blades, but have observed consistent oblanceolate scars (not slit) on leaf cushions and abscised leaves in the matrix. Therefore, we believe that leaves on these axes of *M*. *chaohuensis* had abscised, forming leaf scars on leaf cushions.

Although the terms “leaf cushion” and “leaf scar” have been precisely defined, they are still used indiscriminately at times. Especially, the term “leaf cushion” has been frequently used to describe species or specimens with persistent leaves [3, 37, 38]. To avoid confusion, we suggest more strict correspondences with these terms. “Leaf cushion” should constantly indicate the bulge on the axis whose leaf blade had abscised before been buried in the sediments, it usually bears a true “leaf scar”. “Leaf base” should be used to describe specimens with persistent leaves. When the leaf broke by exogenic force, a “false leaf scar” may be formed on the leaf base. In some conditions, a plant has both thick axes with leaves having abscised and slender axes bearing persistent leaves, e.g., *Minostrobus* (this study) and *Changxingia* Wang [20]. We propose that “leaf cushion” and “leaf base” can be used to describe a single species in order to precisely figure out the different parts of the plant.

### Growth habit

*Minostrobus chaohuensis* has been interpreted as a small herbaceous lycopsid [6], or regarded as “a distal shoot of pseudo-herbaceous or arborescent lycopsids” [8]. In this study, the widest stem is up to 55 mm in diameter (see Description). It was proposed that the deformation of plants was just took place in the vertical dimension during the diagenetic process [39], which has been proved by experiment [40]. Therefore, the size of plants didn’t change horizontally and this widest compression axis represents a stem 55 mm in diameter, approaching that of arborescent lycopsid *Sublepidodendron songziense* (55–70 mm) [3].

Wrinkles on the interareas among leaf cushions are generally interpreted as the result of secondary growth of axes [41, 42]. In *M*. *chaohuensis*, this structure at least occurred on stems and branches thicker than 10 mm, represents the distribution of secondary tissue. Different with this plant, distribution of secondary tissue in pseudoherbaceous and shrubby lycopsids are always very restricted [38], e.g., in rhizomorph, stem and primary branches of *Oxroadia* Alvin [43–45], in rhizomorph and stem base of *Paurodendron* Fry [46–48] and *Chaloneria* Pigg and Rothwell [49].

In arborescent lycopsids, the secondary xylem is not as thick as that in some euphyllophyte trees, but the secondary cortex (periderm) provides the major mechanical support [1, 2, 50]. Therefore, it has been proposed that the proportion of cortex area to axis area in cross section can be used as an index of the growth architecture of the lycopsids [51]. The cortex proportion of *M*. *chaohuensis* is estimated to be 88.7%, exceeding the value of pseudoherbaceous lycopsid *Paurodendron* (75.2%—80.0%), but approaching that of arborescent *Sublepidodendron* (90.7%) [51].

The above evidences suggest that *Minostrobus chaohuensis* is similar to arborescent lycopsids rather than pseudoherbaceous and shrubby species in stem size, secondary tissue and cortex proportion. Therefore, *M*. *chaohuensis* is probably an arborescent lycopsid.

### Whole-plant knowledge and evolutionary significance

Till now, *Minostrobus chaohuensis* has been studied several times [6–8]. Morphology and anatomy of the vegetative axis, both kinds of strobili and spores are now clearly known. Although the root system has not yet been found, we assume that this plant may have stigmarian-type rhizomorph, like other Dichostrobiles members such as *Sublepidodendron* and *Lepidodendron* [3, 52]. *M*. *chaohuensis* is suggested to be a tree-like lycopsid, with a stem at least 55 mm in diameter and multi-dichotomous branching system. The plant is possibly monoecious, with mega- and micro-sporangiate strobili attached to the apex of vegetative axes, bearing *Lagenicula*-type megaspores and *Lycospora*-type microspores [7, 8].

The earliest isoetaleans with monosporangiate strobili have been described from the Late Devonian: *Lepidostrobus xinjiangensis*, *Changxingia longifolia*, *Sublepidodendron songziense* and *Minostrobus chaohuensis*. The latter two species are comparatively more completely known plants and have been proved to be arborescent. The presence of *Sublepidodendron* and *Minostrobus* demonstrates that tree-like Dichostrobiles members already diversified from the bisporangiate-strobilate ancestor and diversified in the Late Devonian. Among rhizomorphic lycopsids, the multi-dichotomous branching system of *Minostrobus* also occurred in the highly derived group Lepidodendraceae and primitive pseudoherbs *Oxroadia* and *Paurodendron*. As DiMichele et al. proposed, the branches of these two groups are possibly nonhomologous because they are separated in the phylogenetic tree by lycopsids with lateral branches on the trunk [53]. In view of the relatively derived reproductive structure of *Minostrobus* [7], we suppose that this plant is phylogeneticly close with Lepidodendraceae rather than *Oxroadia* and *Paurodendron*. This study of Late Devonian *Minostrobus* provides new data on the evolution of growth architecture in rhizomorphic lycopsids.
